# Supplementary material for: Emergence and Evolution of OXA-23-Producing ST46Pas-ST462Oxf-KL28-OCL1 Carbapenem-Resistant Acinetobacter baumannii Mediated by a Novel ISAba1-Based Tn7534 Transposon
Source: Antibiotics (Basel). 2023 Feb 16;12(2):396. doi: 10.3390/antibiotics12020396 (PMC9951949; doi:10.3390/antibiotics12020396)
Supplement: Supplementary file 1 [file antibiotics-12-00396-s001.zip › antibiotics-2099292-supplementary.pdf]

Figure S1. Pan-genome analysis of ST46Pas *A. baumannii* strains using Roary.

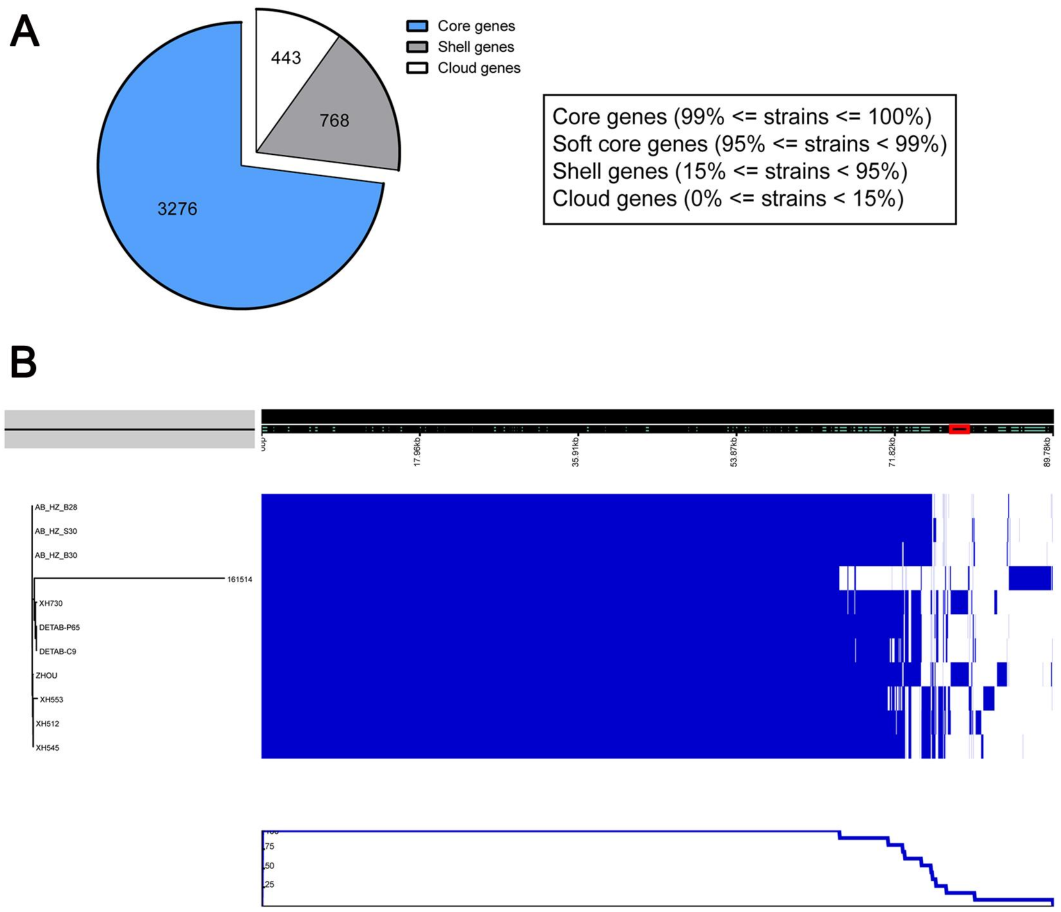

Table S1. Site-specific recombinases XerC/XerD (C/D) and XerD/XerC (D/C) in the chromosome of *A. baumannii* ZHOU isolate

Name	Start	End	Left arm	Center	Right arm	Site
p*dif*-1	89357	89384	ATTTCCGCTAA	TCGGTT	TTATTTTATTG	C|D
p*dif*-2	113932	113959	ATTTAAAATAA	AAATAC	TTATATTACTT	D|C[1]
p*dif*-3	449000	449027	TTTTTACATAA	TAAGTT	TTTTGAAAATT	C|D
p*dif*-4	645347	645374	ATATTTTATAA	TTTATT	GTATTTTAAAT	D|C
p*dif*-5	1402913	1402940	TGTTCGTATAA	TGTATA	TTATGTTAAAT	C|D
p*dif*-6	1435523	1435550	ATTAAATATAA	GTTTAA	TTATGTAATAT	D|C
p*dif*-7	1741796	1741823	AGTTAAAATA A	TATATA	TCATATTAAAA	C|D
p*dif*-8	1909999	1910026	CTTTAAAATAA	GTATTA	TTATGAAGATT	D|C
p*dif*-9	2821899	2821926	GTTAAACCTAA	GAAACC	TTATTTTAAAT	C|D
p*dif*-10	2838943	2838970	TCTCAACATAA	AAATAT	TGAAGTTAAAT	D|C
p*dif*-11	2956941	2956968	AATAAACATA A	ACTGAG	ATATTTTAAAT	C|D
p*dif*-12	3037792	3037819	ATTTATTATAA	ATATAA	TTATTTTATTC	D|C

p*dif*-13	3902192	3902219	ATGTAAGGTAA	ACGGTC	TTCTTCGATAT	C|D
p*dif*-14	3937447	3937474	AGATGGTATAA	ATACTC	TTAAGTTATAT	D|C

[1]p*dif*-2 and p*dif*-3 sites with grey shading were located in each side of *bla*$_{OXA-23}$ segment, respectively.
